# Effects of meta-tetrahydroxyphenylchlorin photodynamic therapy on isogenic colorectal cancer SW480 and SW620 cells with different metastatic potentials

**DOI:** 10.1007/s10103-018-2524-7

**Published:** 2018-05-24

**Authors:** Gulinur Abdulrehman, Kaiyue Xv, Yuhua Li, Ling Kang

**Affiliations:** College of Public Health, Xinjiang Medical University, No. 393, Xinyi Road, Xinyi District, Urumqi, Xinjiang China

**Keywords:** Photodynamic therapy, Meta-tetrahydroxyphenylchlorin, Colorectal cancer, Cell death, Apoptosis

## Abstract

The aim of this study is to investigate the antitumor effects and possible mechanisms of meta-tetrahydroxyphenylchlorin-mediated photodynamic therapy (m-THPC-PDT) on human primary (SW480) and metastatic (SW620) colon cancer cell lines. SW480 and SW620 cells were incubated with various concentrations of m-THPC, followed by photodynamic irradiation. Subcellular localization of m-THPC in cells was observed with confocal laser scanning microscopy (CLSM). Photocytotoxicity of m-THPC in the two cells was investigated by using MTT assay. The flow cytometry was employed to detect the cell apoptosis. The migration and long-term recovery ability were determined by scratch test and colony formation assay respectively. CLSM showed that m-THPC was mainly distributed within the endoplasmic reticulum (ER) and lysosome of SW480 cells and within the lysosome and mitochondria of SW620 cells. m-THPC-PDT induced a dose-dependent and light energy-dependent cytotoxicity in SW480 and SW620 cells. Apoptosis rate was approximately 65 and 25% in SW480 and SW620 respectively when the concentration of m-THPC increased to 11.76 μM. However, the rate of necrotic cells had no significant changes in two cell lines. The colony formation and migration ability of the two cell lines were decreased with m-THPC-PDT treatment in a dose-dependent manner. PDT with m-THPC not only could effectively inhibit cell proliferation and decrease migration ability and colony formation ability, but also could effectively kill SW480 and SW620 cells in a dose-dependent manner in vitro. These results suggest that m-THPC is a promising sensitizer that warrants further development and extensive studies towards clinical use of colorectal cancer.

## Introduction

Colorectal cancer (CRC) is the third most common cancer in males and the second in females worldwide. There are more than 1.2 million new cases and 0.6 million deaths on account of CRC each year [1]. The incidence and mortality rate of CRC is rising year by year in China [2]. The survival rate of CRC patients correlates with tumor stage and the 5-year relative survival is approximately 65%. The 5-year survival rate of patients with advanced disease and unresectable metastatic lesions (stage IV) drops to approximately 5%. Many patients with earlier stage disease (stage II and, in particular, stage III) relapse following surgery and adjuvant chemotherapy treatment. Prognosis is poor for many patients that are diagnosed at late stages where metastasis has occurred [3, 4]. The most common strategy in treatment of CRC is surgical resection prior to chemotherapeutics and radiotherapy. Although the development of a new concept involving hyperthermic intraperitoneal chemotherapy (HIPEC) and cytoreductive surgery has created promising results, these procedures are so invasive that the rate of major morbidity and mortality is extremely high [5, 6]. Therefore, novel and non-invasive approaches for CRC are required.

Photodynamic therapy (PDT) has presented as a promising treatment against various types of cancer, including bladder, esophageal, glioblastoma, and non-melanoma skin cancers [7]. PDT is a “trinity” treatment modality composed of a photosensitizer (PS), light, and molecular oxygen [8, 9]. It is based on the administration of a non-toxic PS, a light sensitive compound with the preferential retention in neoplastic tissue, and subsequent illumination of the cancer lesion with light of appropriate wavelength [10]. As a result, PS is activated by the specific wavelength, generating several reactive oxygen species (ROS) which eventually kill the cancer cells [11, 12]. Meta-tetrahydroxyphenylchlorin (m-THPC) is a second-generation photosensitizer with remarkable photocytotoxicity of tumor cells [11, 13, 14]. m-THPC-PDT has been used in curative treatment for patients with the head and neck cancer [15]. Emerging reports have shown that m-THPC-PDT could be a promising application for the treatment of other malignant neoplasm, such as the liver, oral cavity, and oropharynx cancer [16, 17]. The cell lines SW480 and SW620, derived from a surgical specimen of a primary colon adenocarcinoma and a lymph node metastasis of the same patient respectively, are a useful patient-matched model for studying colorectal cancer treatment in vitro [18, 19]. In the present study, the intracellular distribution of m-THPC was observed first, and then cytotoxicity, apoptotic cell death, and cell biological behaviors of m-THPC-mediated PDT in SW480 and SW620 cell lines were investigated. The results show the effectiveness of m-THPC-PDT in killing human primary and metastatic colorectal cancer cell lines.

## Materials and methods

### Cell culture

The human colon carcinoma cell lines SW480 and SW620 were purchased from Nanjing KGI Biotechnology Co., Ltd., Nanjing, Jiangsu, China (obtained from American Type Culture Collection). The two cell lines were grown in Leibovitz’s L-15 medium supplemented with 10% fetal bovine serum (FBS) (Gibco, Grand Island, NY, USA), 2 mmol/l glutamine, 100 U/ml penicillin, and 100 μg/ml streptomycin (Thermo Scientific, Waltham, MA, USA), in a 37 °C incubator without CO_2_. Cells were passaged every 2–3 days and protected from light constantly after m-THPC treatment.

### m-THPC-PDT

**Fig. 1:**
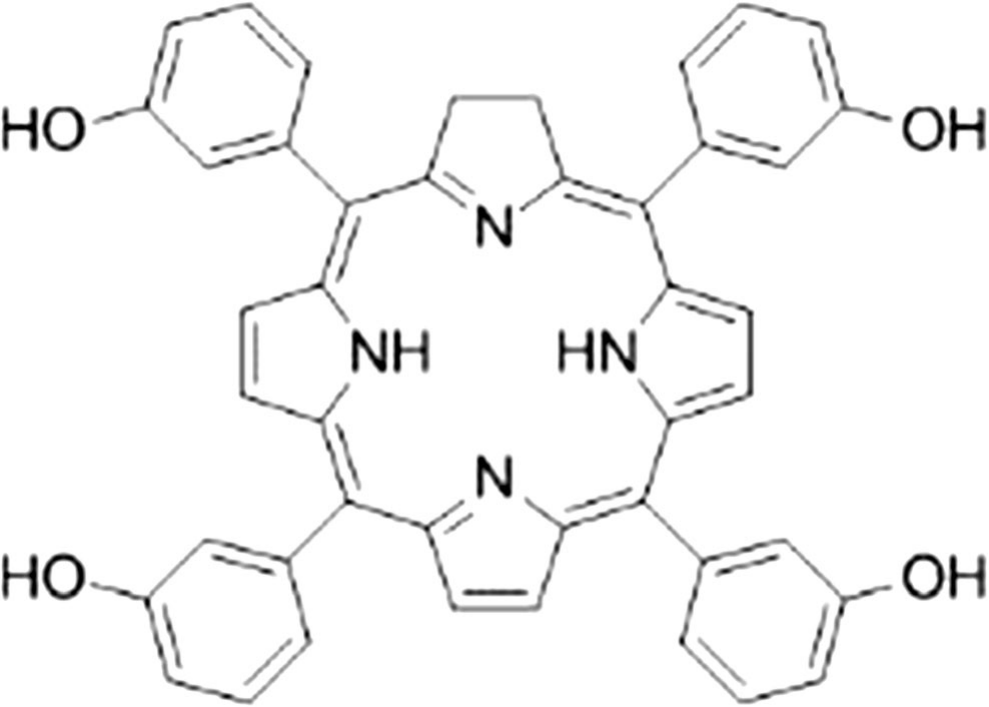
Molecular structural formula of m-THPC

m-THPC was purchased from Frontier Scientific Inc. (Logan, UT, USA), and its molecular structural formula is shown in Fig. 1. m-THPC was dissolved in methanol (2.94 mM stock solution) and stored at − 20 °C in dark. Working solutions were prepared fresh at various concentrations (e.g., 0, 0.18, 0.37, 0.74, 1.47, 2.94, 5.88, and 11.76 μM) and activated by a semiconductor laser (the Key Laboratory of Optoelectronic Technology, Northwestern University, Xi’an, Shaanxi, China) at the light wavelength of 650 nm, a laser power density of 32 mW/cm^2^, and different laser energy densities of 0, 1.5, 3.0, and 6.0 J/cm^2^.

### m-THPC subcellular localization

To confirm the intracellular localization of m-THPC in SW480 and SW620 cell lines, confocal laser scanning microscopy (CLSM) was used. Cells were plated in the confocal laser dish at a density of 1 × 10^4^ cells/ml and incubated for 24 h. Cells were washed with phosphate buffer saline (PBS; in mM, 137 NaCl, 2.7 KCl, 10 Na_2_HPO_4_, and 1.8 KH_2_PO_4_ [pH 7.4]) and incubated with m-THPC at a concentration of 0.74 μM for 8 h. After incubation, plates were washed with PBS and then labeled with 1 μmol/l ER-Tracker^™^ Green, 50 nmol/l LysoTracker Deep Red, and 100 nmol/l MitoTracker Green FM (all from Life Technologies, Gaithersburg, MD, USA) respectively in dark at 37 °C for 30 min. The intracellular fluorescence of the m-THPC, endoplasmic reticulum (ER), lysosome, and mitochondrion was observed using CLSM (Leica, Heidelberg, Germany) with emission wavelengths of 660, 511, 405, and 516 nm respectively.

### Cell viability assay

SW480 and SW620 cells (6 × 10^3^ cells per well) were incubated with different concentrations of m-THPC (0–11.76 μM) in 96-well plates for 8 h. Cell viability was assessed by MTT colorimetric assay. After 24-h culture, these cells were incubated with 20 μl of 5 mg/ml MTT (Sigma, St. Louis, MO, USA) for 4 h under culture conditions. Thereafter, violet-blue formazan crystals were dissolved using 150 μl of DMSO (Sigma) and absorbance measured at 570 nm using a microplate reader (BioRad, Hercules, CA, USA). The percentage of cell survival rate was calculated using the following equation: cell survival rate (%) = 1 − (OD_control_ − OD_treatment_)/(OD_control_ − OD_medium_) × 100%. The half-maximal inhibitory concentration (IC_50_) was calculated using SPSS version 19.0.

### Measurement of cell apoptosis

Flow cytometry (FCM) was employed to analyze cell apoptosis following Annexin V FLUOS Staining Kit (Roche, Basel, Switzerland) according to the manufacturer’s protocol. SW480 and SW620 cells were seeded at a density of 5 × 10^4^ cells/ml in the 6-well plates and incubated for 24 h to allow adherence. Twenty-four hours after m-THPC-PDT treatment, cells were harvested with trypsin enzyme-digesting technique and pelleted by centrifuging at 1000 rpm for 5 min at 4 °C. Cells were resuspended in 100-μl binding buffer, to which Annexin V and propidium iodide (PI) 20 μl were added. Cells were incubated for 30 min in dark, and the normal (intact) cells with low Annexin V and low PI staining, early apoptotic cells with high Annexin V and low PI staining, and necrotic cells with high Annexin V and high PI staining were analyzed by flow cytometer (Beckman Coulter, Miami, FL, USA).

### Detection of colony-forming ability

Plate colony assay was used to determine the cell recovery ability. SW480 and SW620 cells were seeded at a density of 200 cells per well into 24-well plates. After m-THPC-PDT treatment with 6.0 J/cm^2^ of the light energy, cells were cultured continuously for 2 weeks. The colonies were formed, fixed with 5 ml methyl alcohol for 5 min, and stained with Giemsa stain for 30 min. The number of clone, which contained more than 50 cells, was observed through optical microscope (Olympus, Tokyo, Japan). The colony-forming efficiency was calculated using the following equation: colony-forming efficiency (%) = (cloning number/inoculation cell number) × 100%.

### Wound scratch assay

To assess the cellular migration ability after m-THPC-PDT treatment, wound scratch assay was carried out. Cells were seeded in a 24-well plate at a density of 2 × 10^5^ cells/ml (SW480) and 3 × 10^5^ cells/ml (SW620) respectively and cultured for 24 h. Cells were intubated with m-THPC (0–1.47 μM) for 8 h and then irradiated with 6.0 J/cm^2^ light. Ten-microliter sterile pipette tip was used to create the linear scratch. After treatment with m-THPC-PDT, the cells were washed with PBS and further incubated in a complete medium for 48 h. The scratch wound healing was observed under microscope.

### Statistical analysis

Statistical analysis was performed using SPSS version 19.0. The presented data were expressed as mean ± SD of three independent experiments. Two-tailed Student’s *t* test was used to analyze differences between controls and treated samples, and ANOVA was used for the multiple comparisons. Differences in values were stated as significant if the *P* value was less than 0.05.

## Results

### Subcellular localization of m-THPC in SW480 and SW620 cells

**Fig. 2:**
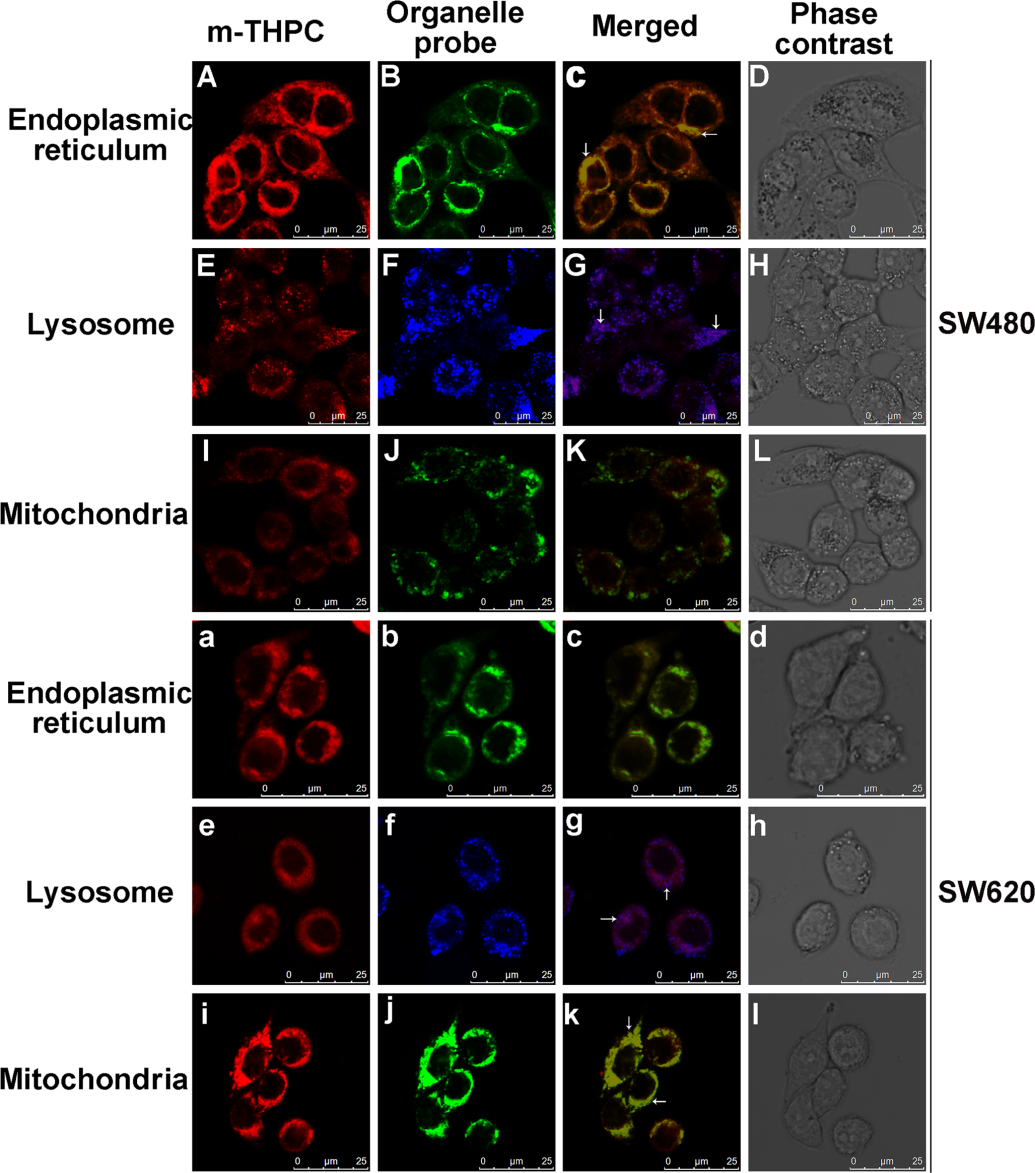
Subcellular localization of m-THPC in SW480 (A–L) and SW620 (a–l) cells. Cells were incubated with 0.74 μM m-THPC for 8 h, then stained with 1 μmol/l endoplasmic reticulum marker (A–D: SW480, a–d: SW620), 50 nmol/l lysosome marker (E–H: SW480, e–h: SW620), and 100 nmol/l mitochondrion marker (I–L: SW480, i–l: SW620) at 37 °C for 30 min. Photographs were taken by confocal laser scanning microscopy. A, E, I, a, e, and i: m-THPC autofluorescence (red); B and b: endoplasmic reticulum marker (green); F and f: lysosome marker (blue); J and j: mitochondrion marker (green); C, G, K, c, g, and k: merged of A and B, E and F, I and J, a and b, e and f, and i and j, respectively; D, H, L, d, h, and l: phase contrast

The CLSM was applied to monitor the subcellular localization of m-THPC in SW480 and SW620 cells. ER-Tracker^™^ Green, LysoTracker Deep Red, and MitoTracker Green FM were used as endoplasmic reticulum, lysosome, and mitochondrion molecule markers respectively. Confocal micrographs of SW480 and SW620 cells incubated with m-THPC and stained with the panel of three different organelle markers are presented in Fig. 2. As shown in Fig. 2, m-THPC exhibited a fluorescence distribution remained only cytoplasmic compartments with no dye detectable in the nucleus in two cell lines (Fig. 2(A, E, I, a, e, i)). In SW480 cells, the intracellular distribution of m-THPC overlapped with that of the ER-Tracker^™^ Green and LysoTracker Deep Red probe which indicates their colocalization in the endoplasmic reticulum and lysosome (Fig. 2(C, G)). However, no colocalization of m-THPC and MitoTracker Green FM was observed within the mitochondria (Fig. 2(K)). The images in Fig. 2(c, g, k) demonstrated that colocalization of m-THPC and organelle probes in SW620 cells occurs mainly in lysosome and mitochondria.

### Photocytotoxicity of m-THPC in SW480 and SW620 cells

**Fig. 3:**
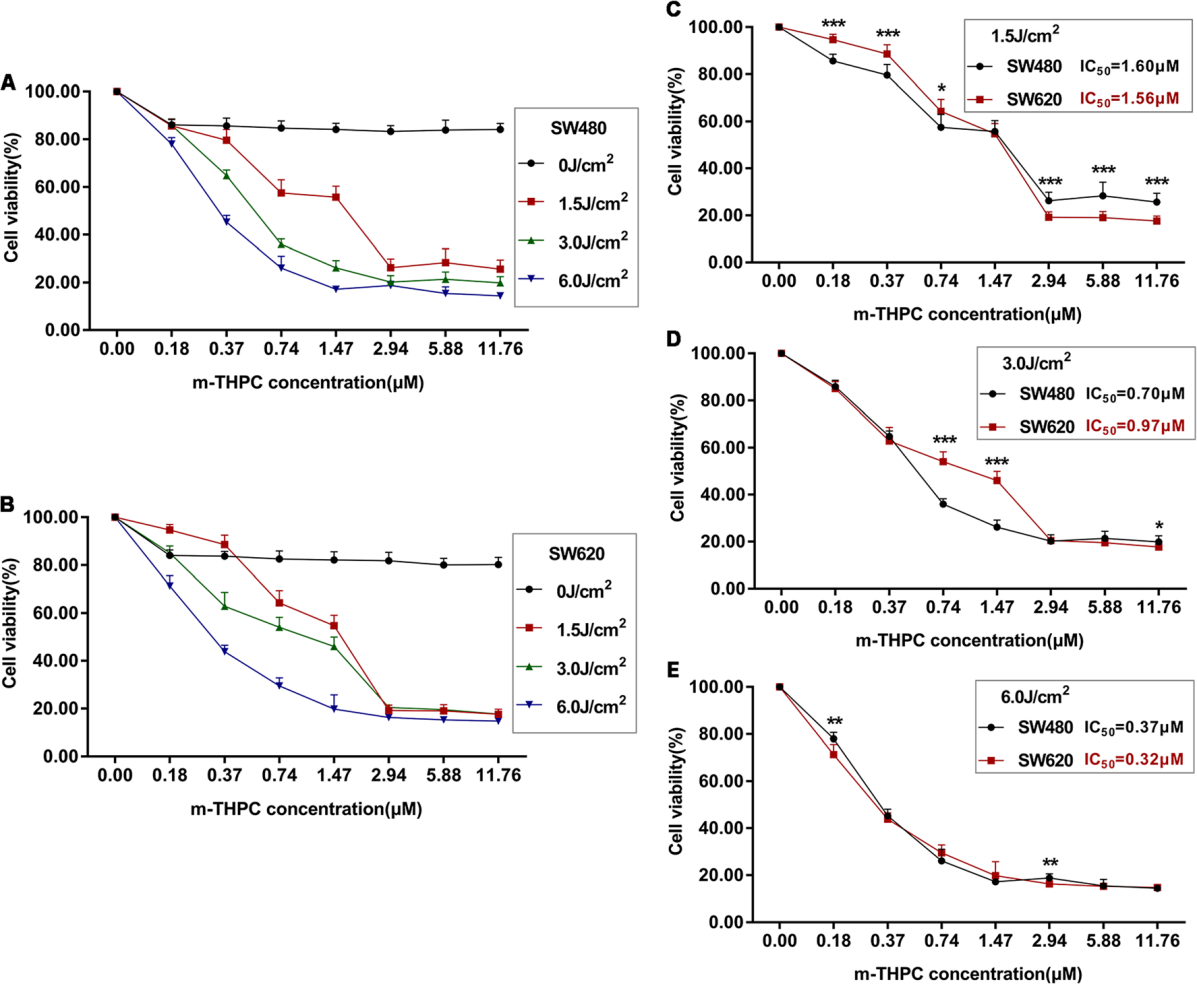
Cell viability of SW480 and SW620 determined by MTT assay after m-THPC-PDT. Two cell lines were incubated with 0–11.76 μM m-THPC for 8 h and then irradiated with 0–6.0 J/cm^2^ light dose. The percentage of cell viability was shown in dose-response curve even 24 h after laser irradiation (**a** SW480, **b** SW620). Comparisons of cell viability in two cell lines with m-THPC at the same concentration and different light doses were demonstrated in **c**, **d**, and **e** (**c** 1.5 J/cm^2^, **d** 3.0 J/cm^2^, and **e** 6.0 J/cm^2^). All results are expressed as the mean ± SD of triplicate determinations from three independent experiments. *P < 0.05, **P < 0.01, ***P < 0.001 compared to the two cell lines

To examine the cytotoxicity effects of m-THPC-PDT on the SW480 and SW620 cells, the cell viability assay was performed at 24 h after PDT treatment. The cells were incubated in different doses of m-THPC (0, 0.18, 0.37, 0.74, 1.47, 2.94, 5.88, and 11.76 μM) for 8 h and exposed to red light from a semiconductor laser with various light energies (0, 1.5, 3.0, and 6.0 J/cm^2^). The experimental results are shown in Fig. 3. m-THPC with irradiation caused a dose-dependent and light energy-dependent cytotoxicity in SW480 and SW620 cells (Fig. 3a, b). No apparent cell death was observed with m-THPC-PDT at a dose of 0.18 and 0.37 μM and a light energy of 1.5 J/cm^2^. Percentage of cell viability was dramatically decreased (approximately from 60 to 20%) with increasing concentration from 0.74 to 2.94 μM of m-THPC accompanied with the light energy from 3.0 to 6.0 J/cm^2^. Change of cell viability was not obvious at a dose of 2.94–11.76 μM of m-THPC with various light energies. Cell survival rate of SW480 and SW620 cells was compared after m-THPC-PDT. The comparison was shown in Fig. 3c–e. Following the PDT with 6.0 J/cm^2^ of the light energy, the difference of cell viability in two cell lines was not evident at the examined concentration of m-THPC. The most significant difference of cell viability was observed at 1.5 J/cm^2^ of the light energy. In two cells, the IC_50_ of m-THPC at 24 h after PDT was decreased in light dose-dependent manner (1.5 J/cm^2^: 1.60 and 1.56 μM for SW480 and SW620 respectively; 3.0 J/cm^2^: 0.70 and 0.97 μM for SW480 and SW620 respectively; 6.0 J/cm^2^: 0.37 μM and 0.32 μM for SW480 and SW620 respectively).

### m-THPC-PDT induces apoptosis in SW480 and SW620 cells

**Fig. 4:**
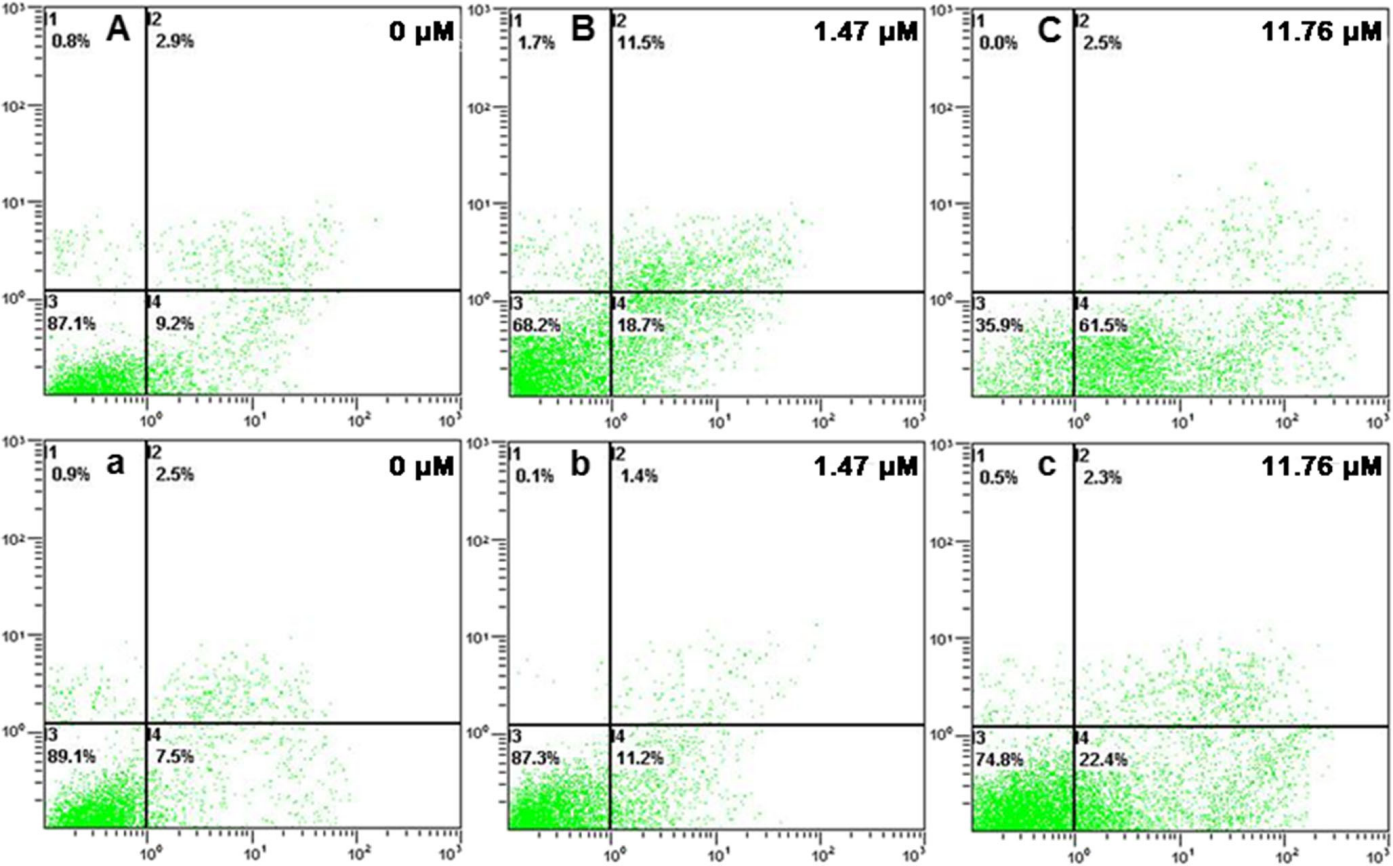
Apoptosis assay using flow cytometry of SW480 and SW620 after m-THPC-PDT treatment. Cells were treated with 0–11.76 μM m-THPC, and Annexin V-fluorescein isothiocyanate (FITC)/propidium iodide (PI) staining was performed after m-THPC-PDT. Representative flow cytometry figures are shown in A–C (SW480) and a–c (SW620)

Apoptotic and necrotic cell deaths induced by PDT have been reported [8, 10, 20, 21]. In order to illustrate the cell death pathways involved in SW480 and SW620 cells treated with m-THPC-PDT, apoptosis was examined by Annexin V and PI staining as detected by flow cytometry. After incubated with m-THPC (0–11.76 μM) for 8 h and then irradiated (0 or 6.0 J/cm^2^), cells were trypsinized and stained with Annexin V and PI 24 h after laser irradiation. As shown in Table 1, the percentage of apoptotic cells in both cell lines was not changed significantly with irradiation or m-THPC only (*P* > 0.05), whereas cells treated with m-THPC-PDT resulted in dose-dependent increase in the percentage of apoptotic cells in both cell lines (*P* < 0.05). As shown in Fig. 4, increasing the m-THPC concentration to 11.76 μM resulted in approximately 65 and 25% apoptotic cells in SW480 (Fig. 4(A–C)) and SW620 (Fig. 4(a–c)) respectively. However, the rate of necrotic cells had no significant changes in two cell lines.

**Table 1:** Apoptosis rate of SW480 and SW620 cells after m-THPC-PDT (%)

m-THPC concentration (μM)	SW480		SW620	
	No irradiation	Irradiation	No irradiation	Irradiation
0	12.70 ± 0.85	12.80 ± 1.48	8.37 ± 1.94	8.37 ± 1.46
0.18	12.83 ± 0.70	17.00 ± 1.31	8.67 ± 1.05	8.63 ± 0.94
0.37	12.83 ± 0.55	21.33 ± 1.62	8.07 ± 3.12	6.80 ± 0.60
0.74	13.10 ± 0.40	29.97 ± 1.56	8.67 ± 2.41	10.03 ± 3.42
1.47	13.10 ± 0.60	37.73 ± 3.62	7.43 ± 0.15	8.80 ± 0.56
2.94	13.20 ± 0.79	54.10 ± 4.94	7.90 ± 0.26	12.07 ± 0.38
5.88	13.03 ± 1.62	66.47 ± 2.61	8.83 ± 2.39	22.80 ± 0.44
11.76	13.33 ± 0.61	65.53 ± 3.78	8.70 ± 2.07	23.77 ± 2.44
*F*	0.19	164.59	0.19	51.57
*P*	0.98	0.00	0.98	0.00

### Decrease of colony formation induced by m-THPC-PDT

The effect of m-THPC-PDT on the colony formation ability of SW480 or SW620 cells was analyzed using the colony formation assay. Cells were cultured continuously in L-15 medium containing 10% FBS at 37 °C for 2 weeks after m-THPC-PDT treatment. Individual clones with more than 50 cells were counted. The colony formation assay indicated that two cell lines formed more and more low numbers of colonies with an increase of m-THPC concentration (Table 2). About 50% decrease in colony-forming efficiency was observed in the SW480 and SW620 cells treated with m-THPC at 2.94 and 0.37 μM separately. The colony-forming efficiency of SW480 and SW620 treated with m-THPC-PDT at 11.76 and 2.94 μM was dropped to 0.

**Table 2:** Effects of m-THPC-PDT on the colony-forming efficiency of SW480 and SW620 cells (%)

m-THPC concentration (μM)	SW480	SW620
0	7.89 ± 1.83	22.61 ± 1.45
0.18	6.89 ± 0.82	18.89 ± 1.76
0.37	6.11 ± 1.31	10.94 ± 1.79
0.74	6.83 ± 1.67	9.56 ± 0.68
1.47	5.72 ± 0.69	0.44 ± 0.39
2.94	3.61 ± 0.65	0 ± 0
5.88	1.61 ± 0.85	0 ± 0
11.76	0.00 ± 0.00	0 ± 0
*F*	68.554	599.054
*P*	0.00	0.00

### Cell migration inhibition caused by m-THPC-PDT on SW480 and SW620 cells

**Fig. 5:**
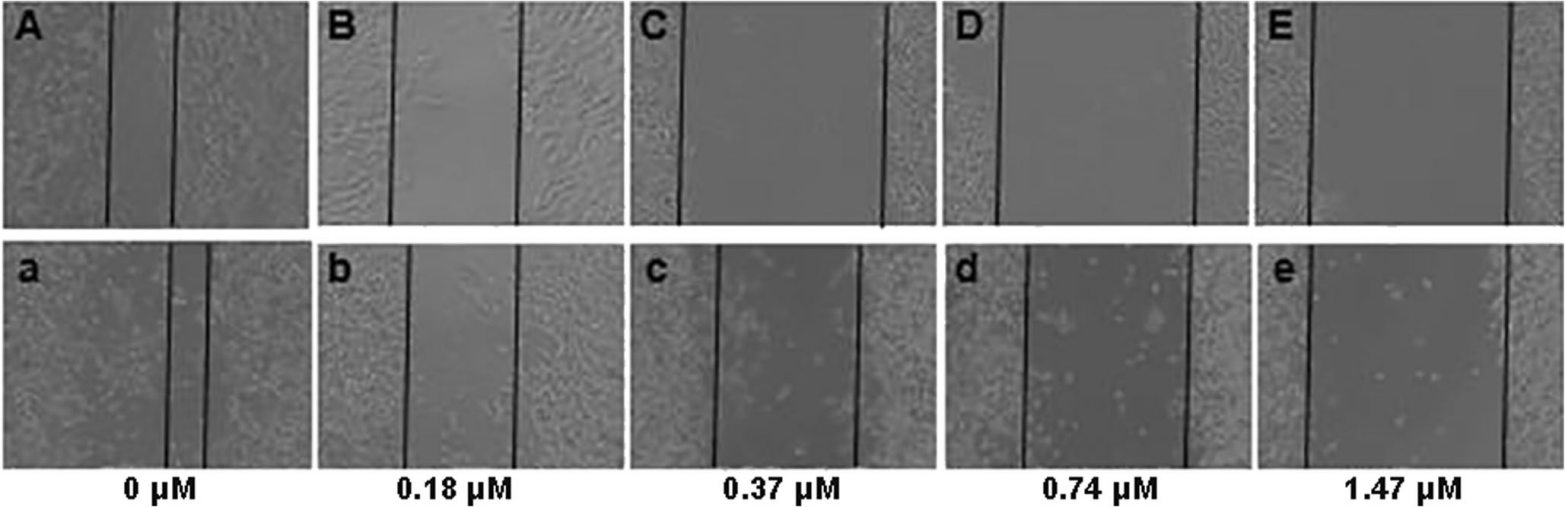
Cell migration ability of SW480 and SW620 after m-THPC-PDT treatment using scratch wound assay. The effects of different concentrations of m-THPC-PDT on cell migration are shown in A–E (SW480) and a–e (SW620). Phase contrast images were taken using the Olympus microscope at 48 h time point after m-THPC-PDT

Cell migration was assessed using scratch wound assay. Cells were plated in a 24-well plate at a density of 2 × 10^5^ cells/ml (SW480) and 3 × 10^5^ cells/ml (SW620) respectively and wounded by scratching with a sterilized white tip after attachment. Following the treatment of m-THPC-PDT, the cells were washed with PBS and further incubated in a complete medium for 48 h. The two cells with the m-THPC-PDT exhibited reduced migration into the wound in a dose-dependent manner (Fig. 5). At the concentration of 0.37 and 0.74 μM m-THPC, m-THPC-PDT had a stronger inhibitory ability to the migration of SW480 than that of SW620 (Fig. 5(C, D, c, d)). However, the effect was the same at the 1.47 μM m-THPC-PDT (Fig. 5(E, e)). Cells untreated and m-THPC alone groups showed the same migration ability as the cell treated with the 0 μM m-THPC-PDT (data not shown).

## Discussion

PDT, a non- or minimally invasive therapeutic approach, is based on tumor-specific accumulation of a photosensitizer followed by its activation using an appropriate wavelength of laser light, which ultimately causes the generation of highly ROS within the tumor cells and induces tumor cell death [8, 14, 22, 23]. PDT is almost pain-free and more convenient for both patients and clinics and has been applied to gastrointestinal tumors [24–26], including colon and rectal cancers. Because PDT does not cause essential effect on connective tissues, the anatomical integrity of hollow organs such as the colon can be preserved in patients undergoing PDT [27, 28].

m-THPC, a second generation PS, is considered a particularly potent PS activated at 652 nm, with a residual photosensitivity of only 2 weeks [29, 30]. m-THPC was used with curative purposes in the treatment of early-stage head and neck cancers and has been given approval for treatment of advanced head and neck cancers [15, 29]. As reported previously, preclinical studies (in vitro photodynamic studies and animal experiments) and clinical trials of m-THPC-PDT to colorectal cancer showed positive and promising results [31]. To investigate potential roles of m-THPC-PDT in colon cancer, the two isogenic colorectal cancer cell lines SW480 and SW620 were used in present study. The primary cancer cell line SW480 was derived from a Dukes’ stage B colon carcinoma, a poorly differentiated (grade 4) CRC invading the muscularis propria, and the metastatic SW620 variant was derived from the same patient’s tumor metastasized to the lymph node [18]. The intracellular localization of m-THPC in SW480 and SW620 was observed by using CLSM firstly as it plays a crucial role for the mechanism and outcome of PDT treatment. Our results showed that m-THPC was mainly located within the endoplasmic reticulum and lysosome in SW480 cells and within lysosome and mitochondria in SW620 cells (Fig. 2). Our previous investigation indicated that another PS, chlorin e6 (Ce6), was mainly located in the ER, followed by the mitochondria, with hardly any distribution in the lysosome and nuclei in SW620 cells (data not shown). Previous reports show that the subcellular localization of m-THPC in MCF-7 cells is endoplasmic reticulum and Golgi apparatus [32, 33]. These results illustrated that intracellular localization of photosensitizer varies depending on the cell and photosensitizer type.

On evaluating the photocytotoxicity of m-THPC-PDT in SW480 and SW620 cells, cell viability was evaluated by MTT assay. As the majority of survey results [13, 15, 34–36], light or m-THPC alone did not lead to significant decrease of survival fraction on SW480 and SW620 cells. Cell cytotoxicity of m-THPC-PDT increases with increasing concentration of m-THPC and the intensity of light irradiation. The effect of m-THPC-PDT on SW480 and SW620 cells was concentration- or dose-dependent, and the cell toxicity was positively correlated with m-THPC concentration and light dose. For IC_50_, compared with previous studies, m-THPC showed much higher cytotoxic than ALA-mediated PDT in SW480 and SW620 cells [37]. In addition, a newly developed photosensitizer, glycoconjugated chlorin (H_2_TFPC-SGlc) increased its cellular uptake and antitumor effect by conjugating with sugar chains; thus, H_2_TFPC-SGlc-mediated PDT (IC_50_: 0.52 μM in HT29 and 0.35 μM in HCT116, 16 J/cm^2^) is about 30 times more cytotoxic than talaporfin-mediated PDT in colon cancer cell lines HT29 and HCT116 [38]. It can be seen that the m-THPC’s IC_50_ is close to the IC_50_ of H_2_TFPC-SGlc in different colon cancer cell lines, under the low light dose (6 J/cm^2^) condition. Therefore, m-THPC is a very promising photosensitizer, and trying to synthesize glucoconjugated derivatives of m-THPC to reduce its IC_50_ may improve the anticancer efficacy of m-THPC-PDT for colon cancer.

Cell death induced by PDT is one of the mechanisms of PDT cytotoxicity [8, 39]. In this study, apoptosis, but not necrosis, was induced in two human colon cancer cell lines displaying different metastatic properties. Although the cell viability was almost at the same level, the more apoptosis rate was observed in SW480 cell line than in SW620 in the same PDT condition. We speculated that the different subcellular localizations of m-THPC in two cell lines might cause the difference of apoptosis rate as it inevitably affects its mechanism of action [40]. As previous reports, several modes of cell death, such as apoptosis, necrosis, necroptosis, and/or autophagy, might be involved in PDT treatment of cancer [41–43]. While autophagy acts as a tumor suppressor and/or a tumor promotor in cancer, an increase in autophagy levels can protect cancer cells against apoptosis induced by PDT [20]. So we suspect that the other cell death models like autophagy may cause cell death by inhibiting apoptosis in SW620 cell, while m-THPC mainly located in the lysosomes of SW620 cells. However, the roles of autophagy induced by PDT were diverse between different cells and different photosensitizers [44–46]. The further investigation are required to identify other cell death modes included in SW480 and SW620 cells treated with m-THPC-PDT.

To further assess the effects of m-THPC-PDT on cell recovery and migration ability of SW480 and SW620 cells in vitro, colony formation and wound scratch assay were carried. The data show that m-THPC-PDT treatment significantly reduces SW480 and SW620 colony formation and migration capacity. Since the initial cell viability assays showed a significant decrease in the two cells, we also determined long-term recovery of cells following m-THPC-PDT treatment, using colony-forming assay. Dose-dependent reduction in colony formation was observed in the two cells, where no colonies formed after PDT with 11.76 μM (SW480) and 5.88 μM (SW620) of m-THPC. It indicates that m-THPC-PDT can inhibit cell recovery of SW620 more effectively. In CRC patients, similar to those with other malignancies, metastases are the main cause of cancer-related mortality. Most patients with metastatic CRC have incurable disease [4, 47]. Therefore, we assessed the effects of m-THPC-PDT on cell migration capacity of the two cells. Up to 1.47 μM of m-THPC, the ability of m-THPC-PDT to inhibit the migration of the two cells was different. The results suggest that high concentration of PDT might be better for inhibiting cell migration.

Overall, our results revealed that m-THPC was effectually absorbed by colon SW480 and SW620 cancer cells and localized to different intracellular organelles in the two cell lines, including the endoplasmic reticulum, mitochondria, and lysosomes. m-THPC-mediated PDT could significantly kill cells, induce cell apoptosis, and inhibit cell colon formation and cell migration. Though further study is required to confirm the efficacy of the photosensitizer m-THPC in vivo, the present results may shed light on photodynamic therapy with m-THPC which may be a useful photosensitizer for the treatment of colon cancer.
